# Heritable variation at the chromosome 21 gene *ERG* is associated with acute lymphoblastic leukemia risk in children with and without Down syndrome

**DOI:** 10.1038/s41375-019-0514-9

**Published:** 2019-07-11

**Authors:** Adam J. de Smith, Kyle M. Walsh, Libby M. Morimoto, Stephen S. Francis, Helen M. Hansen, Soyoung Jeon, Semira Gonseth, Minhui Chen, Hanxiao Sun, Sandra Luna-Fineman, Federico Antillón, Verónica Girón, Alice Y. Kang, Ivan Smirnov, Xiaorong Shao, Todd P. Whitehead, Lisa F. Barcellos, Kent W. Jolly, Jasmine Healy, Caroline Laverdière, Daniel Sinnett, Jeffrey W. Taub, Jillian M. Birch, Pamela D. Thompson, Maria S. Pombo-de-Oliveira, Logan G. Spector, Andrew T. DeWan, Beth A. Mueller, Charleston Chiang, Catherine Metayer, Xiaomei Ma, Joseph L. Wiemels

**Affiliations:** 1 Center for Genetic Epidemiology, Department of Preventive Medicine, University of Southern California, Los Angeles, CA USA; 2 Department of Neurosurgery, Duke University, Durham, NC USA; 3 Duke Cancer Institute, Duke University, Durham, NC USA; 4 Department of Epidemiology and Biostatistics, University of California San Francisco, San Francisco, CA USA; 5 School of Public Health, University of California Berkeley, Berkeley, CA USA; 6 Division of Epidemiology, School of Community Health Sciences, University of Nevada, Reno, NV USA; 7 Department of Neurological Surgery, University of California San Francisco, San Francisco, CA USA; 8 Division of Chronic Disease, Institute of Social and Preventive Medicine, Lausanne, Switzerland; 9 Children’s Hospital Colorado and University of Colorado, Denver, CO USA; 10 Unidad Nacional de Oncología Pediatrica; and Universidad Francisco Marroquín, Guatemala City, Guatemala; 11 Department of Pediatrics, Kaiser Permanente, Roseville, CA USA; 12 Sainte-Justine University Health Center, Montreal, QC Canada; 13 Division of Hematology/Oncology, Children’s Hospital of Michigan, Wayne State University School of Medicine, Detroit, MI USA; 14 University of Manchester, Manchester, UK; 15 Instituto Nacional de Cancer, Rio de Janeiro, Brazil; 16 Department of Pediatrics, University of Minnesota, Minneapolis, MN USA; 17 Department of Chronic Disease Epidemiology, Yale School of Public Health, New Haven, CT USA; 18 Fred Hutchinson Cancer Research Center, Seattle, WA USA

**Keywords:** Cancer epidemiology, Acute lymphocytic leukaemia, Genetics, Acute lymphocytic leukaemia

## To the Editor

Children of Latino ancestry have ~1.6-fold increased risk of acute lymphoblastic leukemia (ALL) relative to non-Latino white children [1], partly explained by the higher frequency of common heritable ALL risk alleles at *ARID5B*, *GATA3*, and *PIP4K2A* in Latinos [2–4]. However, the etiologies of the increased ALL risk in Latinos have not been fully elucidated. We previously performed a large, multi-ethnic genome-wide association study (GWAS) of childhood ALL, including 3,263 cases of which ~60% were of Latino ethnicity [5]. While we identified two novel risk loci, we did not identify Latino-specific risk loci, unlike a recent report from Qian et al. [6]. We have performed whole-genome imputation of our Latino dataset and combined it with GWAS data from two additional, non-overlapping Latino childhood ALL case-control datasets to identify novel and/or Latino-specific risk loci.

The GWAS meta-analysis included the following: (i) 1,949 ALL cases and 2,120 controls from the California Cancer Records Linkage Project (CCRLP-LAT) study, supplemented with 6464 Kaiser GERA study controls [5]; (ii) 38 cases and 49 controls from a Guatemalan ALL case-control study (GTM); and (iii) 312 cases and 454 controls from the California Childhood Leukemia Study (CCLS) [7] (Supplementary Material). Methods for haplotype phasing, whole-genome imputation, and quality-control of imputed genotypes are described in Supplementary Material. Case-control association analyses were performed separately in each study using logistic regression in SNPTEST V2, adjusting for ten ancestry-informative principal components, calculated separately within each dataset. Within-study genomic inflation factors were low (*λ*_CCRLP_ = 1.034, *λ*_GTM_ = 1.01, *λ*_CCLS_ = 1.025). A fixed-effects meta-analysis was performed, and QQ plots indicated adequate control of type I error and minimal population stratification (*λ*_Meta_ = 1.029) (Supplementary Fig. S1).

**Fig. 1:**
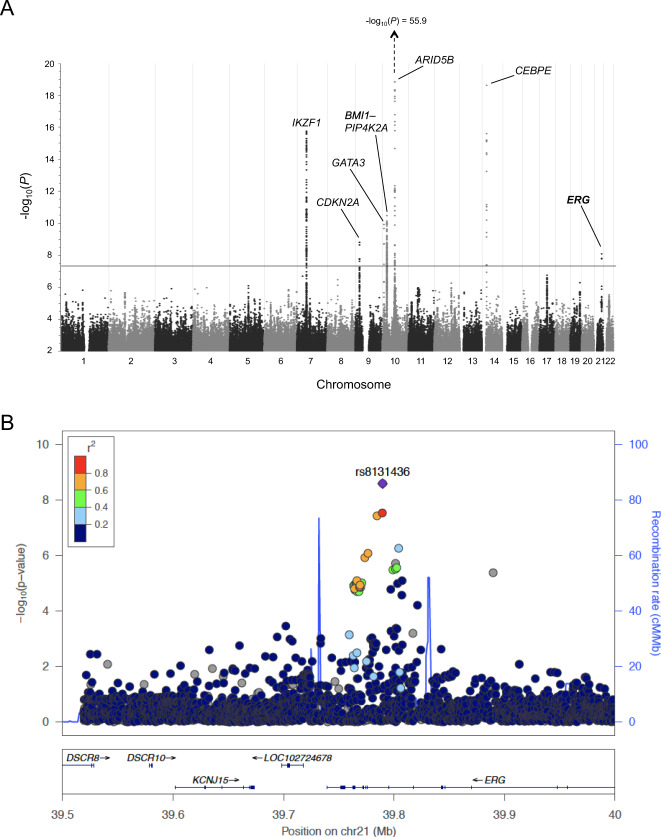
Novel ALL GWAS association locus at *ERG* on chromosome 21. **a** Manhattan plot displaying genome-wide –log_10_(*P*) values from a meta-analysis of three Latino ALL GWAS (*n* = 2,299 cases and 9,087 controls), in the California Cancer Records Linkage Project (CCRLP), the California Childhood Leukemia Study (CCLS), and a Guatemalan ALL study. Grey horizontal line represents the genome-wide significance threshold of *P* = 5.0 × 10^−8^. Genome-wide significant signals were observed at 7 known loci at, in chromosomal order, *IKZF1*, *CDKN2A*, *GATA3*, *BMI1*, *PIP4K2A*, *ARID5B*, and *CEBPE*, as well as a novel locus at *ERG* on chromosome 21. **b** Locus Zoom plot of a ~500Kb region at chromosome 21q22.2 encompassing *ERG*. The −log10(*P*) values were calculated from meta-analysis of the three Latino case-control studies. The ALL association peak is flanked by two recombination hotspot peaks, represented by vertical blue lines

Our GWAS meta-analysis of 2,299 cases and 9,087 controls (Latino only) identified genome-wide significant associations (*P* < 5.0 × 10^–8^) at seven well-established risk loci at *ARID5B*, *CEBPE*, *IKZF1*, *PIP4K2A*, *GATA3*, *CDKN2A*, and *BMI1* [4, 7–10], plus associations (*P* < 5.0 × 10^–4^) at recently identified loci at 17q12/*IKZF3*, 8q24, *LHPP*, and *ELK3* [5, 11] (Supplementary Table S1). We also identified genome-wide significant association at rs8131436 on chromosome 21q22.2, in an intron of the erythroblast transformation-specific (ETS)-related gene (*ERG*) (*P* = 8.76 × 10^–9^; odds ratio [OR] = 1.23; 95% CI: 1.16–1.31) (Fig. 1a). Targeted re-imputation localized the association to an ~100Kb locus between two recombination peaks (Fig. 1b, Supplementary Table S2).

The effect of this locus on ALL risk was recently reported to increase with increasing global Native American (NA) ancestry [6]. Here we examined *local* ancestry at the *ERG* locus (Supplementary Material, Supplementary Fig. S2), and found a larger effect size for rs8131436 in Latinos with ≥1 copy of the NA haplotype (OR = 1.30; 95% CI = 1.15–1.47; *P* = 2.4 × 10^–5^) than in Latinos with zero NA haplotypes (OR = 1.15; 95% CI = 0.98–1.34; *P* = 0.09), further supporting a positive association between NA ancestry and the effect of *ERG* heritable variation on ALL risk. The frequency of NA haplotypes at rs8131436 was slightly higher in cases (42.7%) than controls (40.9%) (Supplementary Fig. S3); however, taking into account the proportion of global NA ancestry, the case-control difference in local NA ancestry at *ERG* was not significant (*P* = 0.44) (Supplementary Table S3).

Next, we investigated whether any *ERG* SNPs were associated with ALL risk in non-Latino whites (*n* = 1184 cases, 3551 controls from CCRLP-EUR) [5]. Of the top 10 *ERG* SNPs in our discovery Latino ALL GWAS meta-analysis, SNP rs2836371 was also associated with ALL in non-Latino whites (*P* = 8.40 × 10^–3^), albeit with a smaller effect size (OR = 1.15, 95% CI: 1.05–1.25) (Supplementary Table S2).

*ERG* is within the Down syndrome (DS) critical region on chromosome 21, and children with trisomy 21 have an ~20-fold increased risk of ALL [12]. Therefore, we explored whether *ERG* variation may contribute to DS-ALL risk. We genotyped rs2836371 (lead SNP across Latino discovery and non-Latino white replication sets) using a Taqman SNP genotyping assay in a Latino case-control set (DS-ALL cases, *n* = 103 and DS non-leukemia controls, *n* = 96) from the International Study of Down Syndrome Acute Leukemia (IS-DSAL, Supplementary Material). Trisomic genotypes were manually clustered to delineate the two heterozygote genotypes (TTC or TCC) (Supplementary Fig. S4). We found that rs2836371 was significantly associated with risk of DS-ALL (*P* = 0.016) with a per-allele OR of 1.44 (95% CI: 1.08–1.96), which was noticeably but non-significantly higher than that in non-DS Latinos (OR = 1.19, Supplementary Table S2) (*P*_*interaction*_ = 0.21). Furthermore, subjects with three risk alleles at rs2836371 (CCC genotype) had a 3.7-fold increased risk of ALL compared to DS subjects harboring no risk alleles (TTT), rather than the 2.99-fold increased risk predicted under an allelic additive model (Table 1, Supplementary Fig. S5). In a smaller set of non-Latino white DS-ALL cases (*n* *=* 83) and DS controls (*n* *=* 78), rs2836371 was not significantly associated with DS-ALL risk (OR = 1.07, 95% CI: 0.77–1.49), reflecting similar inter-ethnic differences in effect size observed in non-DS participants.

**Table 1:** *ERG* SNP rs2836371 genotype frequencies in Latino and non-Latino white Down syndrome (DS) ALL cases and DS non-leukemia controls from the International Study of Down Syndrome Acute Leukemia (IS-DSAL), measured by Taqman SNP genotyping

rs2836371 genotype	Latinos				Non-Latino whites			
	Cases *n* *=* 103 (%)	Controls *n* *=* 96 (%)	*P*-value	OR (95% CI)	Cases *n* *=* 83 (%)	Controls *n* *=* 78 (%)	*P*-value	OR (95% CI)
TTT	26 (25.2)	34 (35.4)	0.016	1.44 (1.08–1.96)	24 (28.9)	26 (33.3)	0.69	1.07 (0.77–1.49)
TTC	33 (32.0)	35 (36.5)			36 (43.4)	30 (38.5)		
TCC	30 (29.1)	22 (22.9)			14 (16.9)	15 (19.2)		
CCC	14 (13.6)	5 (5.2)			9 (10.8)	7 (9.0)		
Risk allele (C) frequency	0.437	0.326			0.365	0.346		
CCC vs. TTT*			0.02	3.66 (1.17–11.47)			0.57	1.39 (0.45–4.32)

*P* values and odds ratios (OR) are calculated using logistic regression tests, assuming the additive model unless specified

*P values and ORs calculated using chi-square tests

Observed inter-ethnic differences in SNP effect size suggest potential interactions with environmental factors, or with additional germline or somatic genetic alterations. Intriguingly, several published GWAS loci for white blood cell (WBC) traits in adults lie ~50Kb downstream of rs2836371 within *ERG* [13]. These SNPs are in very low linkage disequilibrium (LD) with our ALL-associated SNPs, and are positioned on the other side of a strong recombination peak (Supplementary Fig. S6). Novel analysis of selection signals across *ERG* in Latinos revealed no evidence of positive selection for ALL risk SNPs, but identified a strongly significant signal (population branch statistic >99^th^ percentile genome-wide; haplotype statistic >97^th^ percentile) at the downstream WBC trait locus (Supplementary Fig. S6). SNP rs2836426 showed the strongest selection signal (*P* = 2.2 × 10^–4^) and, though in low LD with ALL risk SNP rs2836371 (*D′* = 0.16 in AMR, 1000Genomes), it is in high LD with several WBC trait-associated SNPs (*D′* = 1 in AMR). No direct association was detected between the low-frequency WBC trait-associated SNPs and ALL risk; however, we found marginally significant synergistic interaction between ALL-associated SNP rs2836371 and three perfectly linked WBC trait SNPs (rs80109907, rs7275212, and rs58030288) on ALL risk in Latinos (*P* = 0.079, OR = 2.00) but not in non-Latino whites (*P* = 0.48, OR = 0.78) (Supplementary Table S4), suggesting Latino-specific cooperation between these two independent trait-associated loci in ALL predisposition.

To explore potential functional effects of ALL-associated SNPs in *ERG*, we assessed 32 SNPs with *P* < 5.0 × 10^–5^ in the Latino meta-analysis, of which 19 replicated in the European data (*P* < 0.05). ERG protein is expressed at low levels in lymphoblastoid cell lines, which prevented accurate expression quantitative trait locus (eQTL) analysis within Genotype Tissue Expression (GTEx) or GEUVADIS RNASeq datasets. In silico analyses, using Haploreg, RegulomeDB, UCSC Genome Browser, and Epigenome Browser, revealed no protein-coding variants, nor any obvious functional candidates based on overlap with putative regulatory elements and transcription factor binding sites.

A recently identified ALL tumor subtype, “*DUX4*-rearranged ALL”, is characterized by somatic *DUX4* rearrangements that result in alternative splicing of *ERG* using an alternative start site at “exon 6 alt” [14]. ALL-associated SNPs at *ERG* did not alter known DUX4 binding motifs, and TF-binding motif analysis did not reveal any SNPs creating novel DUX4 binding motifs.

We assessed whether any SNPs overlapped *ERG* exon 6 alt and found that SNP rs2836361, in tight LD with rs2836371 (*R*^2^ = 0.93 and *D*′ = 0.97 in 1000 Genomes individuals of Mexican ancestry; *R*^2^ = 0.99 and *D*′ = 0.99 in Europeans), was located 3 bp upstream of the first exon 6 alt codon (Supplementary Fig. S7). SNP rs2836361 disrupts a strong exonic splicing silencer (ESS), with the risk allele reducing the score of a silencer motif “TCTCCCAA” [15] from 88.1 (TCTGCCAA containing the rs2836361 protective allele) to 70.9 (TCTGTCAA containing the risk allele). This ESS had the highest predicted score within a region encompassing exon 6 alt + /−100bp. Moreover, we found that the rs2836361 risk allele may increase exonic splicing enhancer activity by elevating the RNA recognition motif score for serine/arginine-rich pre-mRNA splicing factor (SRp40). Hence, the rs2836361 risk allele may increase splicing of the non-canonical *ERG* exon 6 alt, conferring dominant negative effects on wildtype ERG and increased risk of ALL. Further analysis is needed to confirm the causal variant at this locus and its functional effects.

In sum, we report the largest GWAS of childhood ALL among Latinos to date, identifying a risk locus at chromosome 21q22.2, encompassing the hematopoietic transcription factor *ERG*. This gene is frequently somatically mutated in ALL, adding to a growing list of genes that both predispose to ALL and drive tumorigenesis following somatic mutations. Insufficient patient data were available to investigate the relationship between *ERG* SNPs and somatic alterations; however, during preparation of this manuscript, Qian *et al*. reported that the *ERG* risk genotype was negatively correlated with somatic *ERG* deletions [6], supporting that the SNP may somewhat mimic effects of somatic loss of *ERG*.

Novel to our study, we replicated the *ERG* association in a case-control study of Down syndrome-ALL; this is the first reported heritable risk factor for DS-ALL, and may inform future risk stratification in this vulnerable population. Current methods to accurately assess trisomic genotypes using SNP arrays are sub-optimal; next-generation sequencing strategies are warranted to elucidate the contribution of heritable variation across chromosome 21 to DS-ALL risk.

Our study highlights the importance of Latino subjects in elucidating the germline genetic architecture of childhood ALL, and suggests that larger sample sizes may reveal additional important susceptibility loci that inform the biology of leukemogenesis.

## Disclaimer

The ideas and opinions expressed herein are those of the author(s) and do not necessarily reflect the opinions of the State of California, Department of Public Health, the National Cancer Institute, and the Centers for Disease Control and Prevention or their Contractors and Subcontractors.

## Supplementary information

Supplemental Material

Supplemental Figures

Table S1

Table S2

Table S3

Table S4
