# Supplementary material for: Heritable variation at the chromosome 21 gene ERG is associated with acute lymphoblastic leukemia risk in children with and without Down syndrome
Source: Leukemia. 2019 Jul 11;33(11):2746–51. doi: 10.1038/s41375-019-0514-9 (PMC6858994; doi:10.1038/s41375-019-0514-9)
Supplement: Supplementary file 1 — Supplemental Material [file 41375_2019_514_MOESM1_ESM.docx]

**Supplemental Methods**

***Study approval***

The study protocol was approved by the Institutional Review Boards at the California Health and Human Services Agency, University of California (San Francisco and Berkeley), Yale University, Washington State, New York State, the Francisco Marroquín University (Guatemala), and of all participating hospitals. For subjects for which dried bloodspot samples were collected from statewide newborn screening biobanks, including from California, Washington, and New York State biobanks (see below), all individuals were born prior to March 18th 2015 and, therefore, were not subject to the "Common Rule" of the federal Newborn Screening Saves Lives Reauthorization Act of 2014, and we did not require informed consent to use these samples for research. Further, all research using bloodspots was performed without the release of personal identification of any study subjects as approved by appropriate federal and state statutes. For subjects in all other studies, written informed consent was obtained from the parents of the participating subjects.

***California Cancer Records Linkage Project (CCRLP)***

Sample acquisition for the CCRLP GWAS of ALL (PIs: Joseph Wiemels and Xiaomei Ma) has been described previously (1). In brief, newborn dried bloodspots (DBS) were obtained for cases and controls from the California Biobank Program, California Department of Public Health (CDPH), Genetic Disease Screening Program. Childhood ALL cases were identified through linkage between the CDPH statewide birth records (years 1982-2009) and the California Cancer Registry (CCR, diagnosis years 1988-2011), excluding ALL cases already recruited into the California Childhood Leukemia Study (see below), with controls randomly selected and matched on year and month of birth, sex, and race/ethnicity (non-Latino white, non-Latino black, Latino (any race), Asian/Pacific Islander, other). We also included additional controls from the Genetic Epidemiology Research on Aging (GERA) study (2). Cases and controls were genotyped on the Affymetrix Axiom World LAT array. Analyses were limited to Latino and non-Latino white CCRLP subjects in this study, and a total of 109 ALL patients with Down syndrome (DS) were excluded from GWAS analyses (1). Immunophenotype data for CCRLP cases was incomplete, therefore our ALL cases include both B- and T-cell ALL. Further, information on cytogenetic subtypes and somatic alterations was not available, precluding tumor subtype-stratified analyses in the GWAS.

***California Childhood Leukemia Study (CCLS)***

Additional Latino ALL cases and controls were included from the California Childhood Leukemia Study (CCLS, PI: Catherine Metayer), an independent California-based case-control study of childhood leukemia that is described in detail elsewhere (3). In brief, patients with childhood leukemia under 15 years of age were identified and rapidly ascertained into the CCLS from California-based pediatric hospitals from 1995 to 2015, with one to two controls matched to each case on child’s date of birth, sex, Latino ethnicity, and maternal race as indicated on the birth certificate record. DNA samples for cases and controls were obtained from newborn DBS from the California Biobank Program Genetic Disease Screening Program. In the current study, we limited GWAS analyses to Latino ALL cases and controls without DS, genotyped on a single genome-wide SNP array platform, the Illumina OmniExpress array (*n*=312 cases and 454 controls), and these were largely representative of the CCLS population as a whole.

***Guatemalan ALL case-control study***

Guatemalan ALL patients and controls were recruited as part of a pilot gene-environment interaction study (PI: Libby Morimoto) leveraging the diverse Hispanic population of Guatemala, comprised primarily of pure indigenous and mixed (mestizo) indigenous-European peoples. A total of 39 children with incident ALL from a tertiary pediatric oncology hospital in Guatemala City, the Unidad Nacional de Oncología Pediátrica (UNOP), and 49 age, sex, and urban vs. rural status-matched controls (identified as healthy siblings of cardiovascular pediatric patients at an adjacent tertiary cardiovascular referral center, Unidad de Cirugía Cardiovascular) were recruited onto study and gave remission peripheral blood (cases) and saliva (controls). DNA was isolated as previously described (1) and specimens were genotyped using the same Affymetrix Axiom array used for the CCRLP subjects. One ALL case was excluded after genotyping due to detectable trisomy 21, leaving 38 cases and 49 controls passing quality-control filters for analysis.

***The International Study of Down Syndrome Acute Leukemia (IS-DSAL)***

The IS-DSAL includes Down syndrome acute leukemia (ALL and acute myeloid leukemia) cases and DS non-leukemia controls collected from several different studies and sources. For this study, we included 190 DS-ALL cases and 174 DS non-leukemia controls of either Latino or non-Latino white ethnicity, determined from birth certificates or self-reported. Of the 190 cases, 102 were identified from 3,234 ALL cases with unknown trisomy 21 status from the CCRLP ALL GWAS (1). In brief, for each genotyped plate the mean chromosome 21 SNP log R ratio (i.e., hybridization intensity) was calculated for each sample. Then, samples with mean chromosome 21 log R ratio >3 standard deviations from the plate sample mean were assigned as trisomy 21. DNA samples were obtained for additional DS-ALL cases from several Childhood Leukemia International Consortium (CLIC) studies (4), including the CCLS (PI: Catherine Metayer) (3) (*n* = 20, non-overlapping with 312 CCLS ALL cases described above), the Washington State Childhood Cancer study (PI: Beth Mueller) (5) (*n* = 18), the UK Childhood Cancer Study (PI: Jillian Birch) (6) (*n* = 8), a Brazilian ALL study (PI: Maria Pombo-de-Oliveira) (7) (*n* = 1), and the Quebec childhood ALL cohort (PI: Daniel Sinnett) (8) (*n* = 3). Additional DS-ALL cases were included from the Children’s Hospital of Michigan (PI: Jeffrey Taub) (*n* = 9), the University of Minnesota (PI: Logan Spector) (*n* = 14), and the New York State Department of Health Newborn Screening program via linkage between the New York State Cancer Registry and the New York State Congenital Malformations Registry (*n* = 15). Neonatal DBS were obtained from DS controls (*n* = 174), without an ALL or acute myeloid leukemia diagnosis by 15 years of age, from the California Biobank Program, through linkage between the California Department of Public Health Genetic Disease Screening Program and the California Cancer Registry. DNA was extracted from neonatal DBS using the Qiagen DNA Investigator blood card protocol.

***SNP genotyping and whole genome imputation in the discovery Latino GWAS***

CCRLP ALL cases and controls and Guatemalan ALL cases and controls were genotyped using Affymetrix Axiom World LAT arrays, as previously described (1). In brief, following quality control filtering, 757,935 polymorphic autosomal SNPs were included in analyses. Genotype data for additional Latino GERA controls, genotyped on the same Affymetrix arrays, were downloaded from dbGAP (Study Accession: phs000788.v1.p2). Subjects with trisomy 21 (Down syndrome) were identified as described above and excluded from the discovery analyses. Samples were screened for cryptic relatedness using 10,000 unlinked SNPs and excluded if identity-by-descent was >0.15. In total, 1949 Latino cases 8584 Latino controls were included in CCRLP association analyses and 38 cases and 49 controls in Guatemalan association analyses.

CCLS Latinos were analyzed as previously described (9). In brief, pre-diagnostic DNA for CCLS samples was extracted from neonatal bloodcards using the QIAamp DNA Mini Kit (QIAGEN) and genotyped using the Illumina Human OmniExpressV1 platform. Genotype reproducibility was verified using 10 duplicate samples with average concordance >99.99%. Samples with genotyping call rates <98% were excluded. Samples with discordant sex information (reported vs. genotyped sex) were excluded. Samples were screened for cryptic relatedness using 10,000 unlinked SNPs and excluded if identity-by-descent was >0.15. SNPs with genotyping call rates <98% were excluded. Any SNP with a Hardy–Weinberg Equilibrium (HWE) *P*<1×10^−5^ in controls was excluded. In total, 312 Latino cases and 454 Latino controls were included in CCLS association analyses.

SNP imputation and association testing was performed separately in CCRLP, Guatemalan, and CCLS case-control datasets. Haplotype phasing was performed with SHAPEIT (version 2.790) (10) and whole-genome imputation was performed using the Minimac3 software (11) with 64,976 human haplotypes from the 2016 release of the Haplotype Reference Consortium used as the imputation reference panel (12). Quality-control procedures were performed as previously described, with poorly imputed variants (INFO scores <0.60 or posterior probabilities <0.90) removed from respective datasets (13). Case-control association analyses were performed separately in each study using logistic regression in SNPTEST V2 using an allelic additive model and genotype dosages, adjusting for ten ancestry-informative principal components (PCs). After adjusting for the first ten PCs and removing 1Mb regions surrounding previously-identified and replicated ALL GWAS lead SNPs (at *IKZF1, ARID5B, CEBPE, CDKN2A, GATA3, PIP4K2A*, *BMI1, IKZF3,* 8q24*, ELK3* and *LHPP*), genomic inflation factors revealed minimal inflation of test statistics in all case-control sets (λCCRLP=1.034, λGTM=1.01, λCCLS=1.025). For meta-analysis, we removed SNPs with minor allele frequency (MAF) <1% in the combined cases, and variants that appeared in fewer than two datasets. A fixed-effects meta-analysis was performed using META to generate the summary GWAS meta-analysis across the three datasets (14).

***Targeted re-imputation of ERG SNPs at chromosome 21q22.2***

Imputation was carried out across the chromosome 21q22.2 region encompassing the association peak at *ERG*, including coordinates 250Kb upstream and downstream of this gene. Imputation of the ~500Kb region was performed using Impute2 v2.3.1 software and its standard Markov chain Monte Carlo algorithm, with default settings for targeted imputation (15) and using 1,000 Genomes Phase 3 haplotypes for the imputation reference panel (16). Poorly imputed SNPs, *i.e.* those with imputation quality (info) scores <0.60 or posterior probabilities <0.90, were excluded. Association statistics for imputed and directly-genotyped SNPs were calculated using logistic regression in SNPTESTv2, using an allelic additive model and probabilistic genotype dosages (17). The effect of individual SNPs on ALL risk was calculated while adjusting for the first 10 PCs from Eigenstrat. Analyses were carried out separately in the three independent Latino case-control studies: i) the CCRLP; ii) the CCLS; and iii) the Guatemalan study. Meta-analysis of results across the three studies was carried out using the program META (14).

***Replication in the European CCRLP case-control set***

For initial replication of genome-wide significant SNPs from the discovery Latino GWAS meta-analysis, we used a set of 1184 cases and 3551 controls of European ancestry from the CCRLP ALL GWAS (1), with genome-wide SNP array data from the Affymetrix Axiom World LAT arrays. Targeted imputation of the chromosome 21q22.2 locus was carried out as above. We tested SNPs with *P*-value < 5.0 x 10^-5^ and minor allele frequency (MAF) >0.01 from the Latino meta-analysis (*N*=32) for association with ALL in CCRLP Europeans, using logistic regression with an allelic additive model and probabilistic genotype dosages, and adjusting for the first 10 PCs from Eigenstrat.

***Replication in multi-ethnic Down syndrome ALL case-control study***

To test for association of heritable variation at *ERG* with risk of ALL in children with DS (DS-ALL), we carried out an allelic discrimination assay on the BioRad CFX384 Real-Time PCR System using a TaqMan SNP genotyping assay for SNP rs2836371 (Thermo Fisher Scientific assay ID: [C___2439755_10](http://www.thermofisher.com/order/genome-database/details/genotyping/C___2439755_10?CID=&ICID=&subtype=)). We assayed 190 DS-ALL cases and 174 DS non-ALL controls. Manual clustering of trisomic SNP genotypes was carried out using BioRad CFX Manager Software V3.1. We excluded 4 subjects that were not well-clustered into one of the four possible genotypes TTT, TTC, TCC, or CCC (*e.g.* **Figure S3**). SNP rs2836371 did not deviate from Hardy-Weinberg equilibrium in either Latino (P=0.39) or non-Latino white controls (P=0.09). Association analysis was carried out separately in self-reported Latinos (103 cases, 96 controls) and non-Latino whites (83 cases, 78 controls) using additive logistic regression tests.

***Local ancestry analysis***

Local ancestry inference was performed on CCRLP Latino cases and controls using RFMix (22), using a reference panel consisting of 99 CEU individuals for European ancestry, 108 YRI individuals for African ancestry, and 30 Native American (NA) ancestry individuals (29 from PEL, 1 from MXL) from 1000 Genomes Project (16). The 30 NA individuals were selected based on having > 90% NA ancestry in an unsupervised analysis of global ancestry of Admixed American (AMR) populations (CLM, MXL, PEL, and PUR) from 1000 Genomes Project (**Figure S2**). We repeated local ancestry inference using a larger reference panel of NA ancestry by including all 1000 Genomes individuals with > 80% estimated NA ancestry, but this did not improve the precision of local ancestry estimation for the NA, and potentially increased the bias in the mis-estimated segment. After estimating local ancestry for each individual, we stratified Latino individuals into whether they carried zero copies or at least 1 copy of the haplotype derived from NA ancestry at the *ERG* locus, and in each group estimated the OR and confidence interval for association of SNP rs8131436 with ALL risk in a logistic regression model, adjusting for age, sex, and 10 principal components using PLINK v1.9.

We also assessed whether local NA ancestry at the *ERG* locus was significantly over-represented in Latino ALL cases than controls after adjusting for the genome-wide (*i.e.* global) NA ancestry. We carried out case-control logistic regression testing the proportion of local NA ancestry in cases versus controls while adjusting for global NA ancestry in a 2Mb region centered on the *ERG* risk SNP rs8131436.

***Selection signal analysis***

To investigate selection signals across the *ERG* region (chr21:39700000-40000000, hg19) in Latinos, we computed two cross-population statistics for detecting positive selection using whole-genome sequencing data from 1000 Genomes Project (16): i) an allelic differentiation statistic known as Population Branch Statistics (PBS) (23); and ii) an extended haplotype homozygosity statistic “cross-population number of segregating sites by length”, or XP-nSL, which is an in-house developed cross-population extension of the nSL statistics (24). Specifically, we examined alleles that showed significant differentiation along the MXL (Mexican Ancestry from Los Angeles USA, 1000 Genomes) branch (chosen as a representative population of Latinos), relative to two reference populations of Asian and African ancestry (CHB and YRI, respectively). For each SNP, an F_ST_ value was estimated between pairs of populations using Weir and Cockerham’s method (25), and PBS was calculated as: $PBS = \frac{T^{MXL, EAS}+T^{MXL, YRI}-T^{EAS, YRI}}{2}$,

where $T = -\log\left( 1-F_{ST} \right)$ is an estimation of the population divergence time. Sites with MAF<0.01 in any pair of populations were excluded.

We also computed the XPnSL statistic to compare haplotype homozygosity between MXL population and East Asian (EAS) populations. EAS super-population included CHB, CHS, CDX, JPT, and KHV populations from 1000 Genomes Project. We only kept bi-allelic SNPs with MAF>0.01 in the union of MXL and EAS populations. XPnSL scores were calculated per SNP, and then standardized to zero mean and unit variance. We then divided the genome into overlapping windows of 100Kb (with a step size of 50Kb). Candidate regions for selection were identified by calculating the fraction of SNPs with |XPnSL| > 2 in each window. Both PBS and XPnSL were calculated using the “scikit-allel” tool [https://scikit-allel.readthedocs.io/en/latest/].

***Interaction analysis with white blood cell trait-associated SNPs at chromosome 21q22.2***

Data on white blood cell (WBC) trait-associated SNPs in *ERG* were downloaded from the NHGRI-EBI GWAS Catalog (<https://www.ebi.ac.uk/gwas/>). We identified four SNPs previously associated with WBC traits at genome-wide significance: rs78762153 (associated with basophil count (26)), rs80109907 (monocyte % of white cells and granulocyte % of myeloid white cells (27)), rs7275212 (basophil count (26)), and rs58030288 (sum eosinophil + basophil counts (27)) (**Table S3**). We assessed statistical interaction between ALL-associated SNP rs2836371 and all four WBC trait-associated SNPs separately in CCRLP Latinos and non-Latino whites, using logistic regression tests including an interaction term and adjusting for 10 ancestry-informative PCs.

***Functional assessment of ERG SNPs***

We initially investigated the functional implications of ALL-associated *ERG* SNPs using HaploReg (28), RegulomeDB (29), the UCSC Genome Browser (30) and the Epigenome Browser (31). We assessed whether any SNPs were expression quantitative trait loci (eQTL) using the Genotype-Tissue Expression (GTEx) Project (32). Additional eQTL analysis was carried out using RNA sequencing (RNA-Seq) data from lymphoblastoid cell lines (LCLs) in the GEUVADIS project (33); however, there were very few sequencing reads across *ERG* in this dataset.

Next, we assessed the overlap of *ERG* SNPs with predicted binding sites for DUX4 protein in two ways: 1) we took 14 predicted DUX binding sites across the *ERG* gene locus identified by chromatin immunoprecipitation sequencing (ChIP-Seq) in Zhang *et al.* (2016) (34), and checked for overlap with the 32 candidate *ERG* SNPs; and 2) we checked whether any SNPs created novel DUX4 binding motifs, by exporting 11bp of sequence upstream and downstream of each SNP using the UCSC Genome Browser (as the DUX4 binding motif is 11bp in length). Using JASPAR (<http://jaspar.genereg.net/cgi-bin/jaspar_db.pl>), we searched for the DUX4 binding site motif in the 23bp total sequence around each SNP, including either the reference or alternate allele.

To assess the effects of SNP rs2836361 on potential splicing activity at *ERG* exon 6 alternate, or *“*exon 6 alt” (34), we inputted the exon 6 alt DNA sequence (chr21:39,764,565-39,764,585) +/- 100bp into the Human Splicing Finder software (<http://www.umd.be/HSF3/4DACTION/input_SSF>) (35), including either the non-risk (G) or risk (A) allele. The Exonic Splicing Enhancer (ESE) Finder software (36) was subsequently used to determine whether SNP rs2836361 affected ESE activity.

**References:**

(1) Wiemels JL, Walsh KM, de Smith AJ, Metayer C, Gonseth S, Hansen HM, et al. GWAS in childhood acute lymphoblastic leukemia reveals novel genetic associations at chromosomes 17q12 and 8q24.21. Nat Commun 2018 Jan 18;9(1):286-017-02596-9.

(2) Banda Y, Kvale MN, Hoffmann TJ, Hesselson SE, Ranatunga D, Tang H, et al. Characterizing Race/Ethnicity and Genetic Ancestry for 100,000 Subjects in the Genetic Epidemiology Research on Adult Health and Aging (GERA) Cohort. Genetics 2015 Aug;200(4):1285-1295.

(3) Metayer C, Zhang L, Wiemels JL, Bartley K, Schiffman J, Ma X, et al. Tobacco smoke exposure and the risk of childhood acute lymphoblastic and myeloid leukemias by cytogenetic subtype. Cancer Epidemiol Biomarkers Prev 2013 Sep;22(9):1600-1611.

(4) Metayer C, Milne E, Clavel J, Infante-Rivard C, Petridou E, Taylor M, et al. The Childhood Leukemia International Consortium. Cancer Epidemiol 2013 Jun;37(3):336-347.

(5) Podvin D, Kuehn CM, Mueller BA, Williams M. Maternal and birth characteristics in relation to childhood leukaemia. Paediatr Perinat Epidemiol 2006 Jul;20(4):312-322.

(6) The United Kingdom Childhood Cancer Study: objectives, materials and methods. UK Childhood Cancer Study Investigators. Br J Cancer 2000 Mar;82(5):1073-1102.

(7) Pombo de Oliveira MS, Koifman S, Vasconcelos GM, Emerenciano M, de Oliveira Novaes C, Brazilian Collaborative Study Group of Infant Acute Leukemia. Development and perspective of current Brazilian studies on the epidemiology of childhood leukemia. Blood Cells Mol Dis 2009 Mar-Apr;42(2):121-125.

(8) Healy J, Belanger H, Beaulieu P, Lariviere M, Labuda D, Sinnett D. Promoter SNPs in G1/S checkpoint regulators and their impact on the susceptibility to childhood leukemia. Blood 2007 Jan 15;109(2):683-692.

(9) Walsh KM, de Smith AJ, Hansen HM, Smirnov IV, Gonseth S, Endicott AA, et al. A Heritable Missense Polymorphism in CDKN2A Confers Strong Risk of Childhood Acute Lymphoblastic Leukemia and Is Preferentially Selected during Clonal Evolution. Cancer Res 2015 Nov 15;75(22):4884-4894.

(10) O'Connell J, Gurdasani D, Delaneau O, Pirastu N, Ulivi S, Cocca M, et al. A general approach for haplotype phasing across the full spectrum of relatedness. PLoS Genet 2014 Apr 17;10(4):e1004234.

(11) Das S, Forer L, Schonherr S, Sidore C, Locke AE, Kwong A, et al. Next-generation genotype imputation service and methods. Nat Genet 2016 Oct;48(10):1284-1287.

(12) McCarthy S, Das S, Kretzschmar W, Delaneau O, Wood AR, Teumer A, et al. A reference panel of 64,976 haplotypes for genotype imputation. Nat Genet 2016 Oct;48(10):1279-1283.

(13) Zhang C, Morimoto LM, de Smith AJ, Hansen HM, Gonzalez-Maya J, Endicott AA, et al. Genetic determinants of childhood and adult height associated with osteosarcoma risk. Cancer 2018 Sep 15;124(18):3742-3752.

(14) Liu JZ, Tozzi F, Waterworth DM, Pillai SG, Muglia P, Middleton L, et al. Meta-analysis and imputation refines the association of 15q25 with smoking quantity. Nat Genet 2010 May;42(5):436-440.

(15) Howie BN, Donnelly P, Marchini J. A flexible and accurate genotype imputation method for the next generation of genome-wide association studies. PLoS Genet 2009 Jun;5(6):e1000529.

(16) 1000 Genomes Project Consortium, Auton A, Brooks LD, Durbin RM, Garrison EP, Kang HM, et al. A global reference for human genetic variation. Nature 2015 Oct 1;526(7571):68-74.

(17) Marchini J, Howie B. Genotype imputation for genome-wide association studies. Nat Rev Genet 2010 Jul;11(7):499-511.

(18) Galanter JM, Fernandez-Lopez JC, Gignoux CR, Barnholtz-Sloan J, Fernandez-Rozadilla C, Via M, et al. Development of a panel of genome-wide ancestry informative markers to study admixture throughout the Americas. PLoS Genet 2012;8(3):e1002554.

(19) Li JZ, Absher DM, Tang H, Southwick AM, Casto AM, Ramachandran S, et al. Worldwide human relationships inferred from genome-wide patterns of variation. Science 2008 Feb 22;319(5866):1100-1104.

(20) Falush D, Stephens M, Pritchard JK. Inference of population structure using multilocus genotype data: linked loci and correlated allele frequencies. Genetics 2003 Aug;164(4):1567-1587.

(21) Walsh KM, Chokkalingam AP, Hsu LI, Metayer C, de Smith AJ, Jacobs DI, et al. Associations between genome-wide Native American ancestry, known risk alleles and B-cell ALL risk in Hispanic children. Leukemia 2013 Dec;27(12):2416-2419.

(22) Maples BK, Gravel S, Kenny EE, Bustamante CD. RFMix: a discriminative modeling approach for rapid and robust local-ancestry inference. Am J Hum Genet 2013 Aug 8;93(2):278-288.

(23) Yi X, Liang Y, Huerta-Sanchez E, Jin X, Cuo ZX, Pool JE, et al. Sequencing of 50 human exomes reveals adaptation to high altitude. Science 2010 Jul 2;329(5987):75-78.

(24) Ferrer-Admetlla A, Liang M, Korneliussen T, Nielsen R. On detecting incomplete soft or hard selective sweeps using haplotype structure. Mol Biol Evol 2014 May;31(5):1275-1291.

(25) Weir BS, Cockerham CC. Estimating F-Statistics for the Analysis of Population Structure. Evolution 1984 Nov;38(6):1358-1370.

(26) Okada Y, Hirota T, Kamatani Y, Takahashi A, Ohmiya H, Kumasaka N, et al. Identification of nine novel loci associated with white blood cell subtypes in a Japanese population. PLoS Genet 2011 Jun;7(6):e1002067.

(27) Astle WJ, Elding H, Jiang T, Allen D, Ruklisa D, Mann AL, et al. The Allelic Landscape of Human Blood Cell Trait Variation and Links to Common Complex Disease. Cell 2016 Nov 17;167(5):1415-1429.e19.

(28) Ward LD, Kellis M. HaploReg: a resource for exploring chromatin states, conservation, and regulatory motif alterations within sets of genetically linked variants. Nucleic Acids Res 2012 Jan;40(Database issue):D930-4.

(29) Boyle AP, Hong EL, Hariharan M, Cheng Y, Schaub MA, Kasowski M, et al. Annotation of functional variation in personal genomes using RegulomeDB. Genome Res 2012 Sep;22(9):1790-1797.

(30) Kent WJ, Sugnet CW, Furey TS, Roskin KM, Pringle TH, Zahler AM, et al. The human genome browser at UCSC. Genome Res 2002 Jun;12(6):996-1006.

(31) Zhou X, Maricque B, Xie M, Li D, Sundaram V, Martin EA, et al. The Human Epigenome Browser at Washington University. Nat Methods 2011 Nov 29;8(12):989-990.

(32) GTEx Consortium. The Genotype-Tissue Expression (GTEx) project. Nat Genet 2013 Jun;45(6):580-585.

(33) Lappalainen T, Sammeth M, Friedlander MR, 't Hoen PA, Monlong J, Rivas MA, et al. Transcriptome and genome sequencing uncovers functional variation in humans. Nature 2013 Sep 26;501(7468):506-511.

(34) Zhang J, McCastlain K, Yoshihara H, Xu B, Chang Y, Churchman ML, et al. Deregulation of DUX4 and ERG in acute lymphoblastic leukemia. Nat Genet 2016 Dec;48(12):1481-1489.

(35) Desmet FO, Hamroun D, Lalande M, Collod-Beroud G, Claustres M, Beroud C. Human Splicing Finder: an online bioinformatics tool to predict splicing signals. Nucleic Acids Res 2009 May;37(9):e67.

(36) Cartegni L, Wang J, Zhu Z, Zhang MQ, Krainer AR. ESEfinder: A web resource to identify exonic splicing enhancers. Nucleic Acids Res 2003 Jul 1;31(13):3568-3571.

**Supplemental Figure and Table Legends:**

**Figure S1 –** Quantile-quantile (Q-Q) plot of observed versus expected *P*-values in the Latino ALL GWAS meta-analysis, following exclusion of 1Mb regions centered on known GWAS hits at *IKZF1, ARID5B, CEBPE, CDKN2A, GATA3, PIP4K2A*, *BMI1, IKZF3,* 8q24*, ELK3* and *LHPP*. Genomic inflation factors revealed minimal inflation of test statistics in the overall meta-analysis (λ_Meta_=1.029).

**Figure S2 –** Plot showing results from global ancestry analysis on Admixed American and other continental populations from 1000 Genomes Project using ADMIXTURE. The 1000 Genomes Project Admixed American populations included: CLM = Colombians from Medellin, Colombia; MXL = Mexican Ancestry from Los Angeles, USA; PEL = Peruvians from Lima, Peru; and PUR = Puerto Ricans from Puerto Rico. Additional population included were: CEU = Utah Residents with Northern and Western European Ancestry; CHB = Han Chinese in Beijing, China; and YRI = Yoruba in Ibadan, Nigeria. Different colored bars correspond to proportions of different ancestries: red = Native American; purple = European; green = African; blue = East Asian; and orange = unexplained.

**Figure S3** – Plot showing the frequency of Native American (NA), European (CEU), and African (YRI) haplotypes at the *ERG* risk locus (SNP rs8131436) in Latino childhood ALL cases (dark grey) and Latino controls (light grey) from the California Cancer Records Linkage Project (CCRLP) study. Local ancestry inference was performed using RFMix. Error bars represent 95% confidence intervals generated by bootstrapping (bootstrap *n* = 1000).

**Figure S4** – Taqman SNP genotyping results for rs2836371 in DS-ALL cases and DS non-leukemia controls, displayed using BioRad CFX Manager Software V3.1.

**A:** Results from one plate demonstrating clear distinction between subjects with the two possible heterozygote genotypes TTC and TCC (green), and between heterozygote subjects and subjects homozygous for genotype TTT (orange) or CCC (blue). RFU: relative fluorescence unit;

**B:** In another plate, distinct clusters for each trisomic genotypic were seen, but three subjects (red crosses) were excluded from subsequent analyses as we were not able to assign their genotypes.

**Figure S5** – DS-ALL risk in Latinos as a function of *ERG* SNP rs2836371 risk allele count. We calculated the expected effect size (“T21 LAT Expected”, blue data points) for each additional copy of the risk allele (*i.e.* 1 vs. 0, OR=1.44; 2 vs. 0, OR=2.07; and 3 vs. 0, OR=2.99) assuming an additive effect and using the per-allele odds ratio = 1.44 as generated from the case-control logistic regression in DS Latinos. This is compared with the observed effect size (“T21 LAT Observed”, orange data points) for each additional risk allele copy in DS Latinos (1 vs. 0, OR=1.23; 2 vs. 0, OR=1.78; and 3 vs. 0, OR=3.66).

**Figure S6 –** Selection signal peak at *ERG*, as calculated using population branch statistics (PBS) in whole-genome sequencing data from 1000 Genomes Project. SNP rs2836426 had the most significant signal for positive selection in the Mexican population (MXL, *P*=2.2x10^-4^), demonstrating the greatest divergence from populations of Asian (CHB) and African (YRI) ancestry. The selection peak overlaps a cluster of GWAS SNPs associated with blood cell traits (light blue shaded box), including four associated with white blood cell (WBC) traits (rs7275212, PMID:21738478; rs78762153, PMID:29403010; rs80109907 & rs58030288, PMID: 27863252) as well as two SNPs associated with red blood cell or platelet traits (rs2836422 & rs2836441 respectively, PMID: 27863252). All four of the WBC trait SNPs are low frequency (MAF=0.01) and in strong linkage disequilibrium with rs2836426 (D′=1), but are in very low linkage disequilibrium with upstream ALL-associated SNPs in *ERG* (pink shaded box). ALL-associated SNPs are separated from the selection peak SNPs and WBC trait-associated SNPs by a strong recombination peak (represented by blue vertical lines).

**Figure S7 –** ALL-associated SNP rs2836361 positioned at the start site of *ERG* “exon 6 alt”, shown in a screenshot from the UCSC Genome Browser. SNP rs2836361 is in tight linkage disequilibrium with SNP rs2836371 (R^2^=0.93 and D′=0.97 in HapMap individuals of Mexican ancestry; R^2^=0.99 and D′=0.99 in Europeans). SNP rs2836361 risk allele T reduces an exonic splicing silencer (ESS) motif score from 88.1 to 70.9, potentially resulting in increased splicing of exon 6 alt. In addition, rs2836361 risk allele T increases the RNA recognition motif score for serine/arginine (SR)-rich pre-mRNA splicing factor SRp40 from 4.15 to 4.51, which may result in increased exonic splicing enhancer (ESE) activity.

**Table S1** – *P*-values and odds ratios for lead SNPs in 11 genomic loci associated with childhood ALL in previously published GWAS, from the Latino GWAS meta-analysis.

**Table S2** – Top 32 SNPs at *ERG* with *P* < 5.0 x 10^-5^ and minor allele frequency > 0.01 in the Latino GWAS meta-analysis, with replication in the non-Latino white CCRLP GWAS and in Latino and non-Latino white DS-ALL cases and DS controls from the International Study of Down Syndrome Acute Leukemia.

**Table S3** – Local admixture mapping results across a 2Mb region centered on the *ERG* locus, using genetic data from Latino ALL cases and controls in the California Cancer Records Linkage Project (CCRLP).

**Table S4** – Interactions between ALL-associated SNP rs2836371 and four white blood cell (WBC) trait-associated SNPs at *ERG*, in Latinos and non-Latino whites, in the California Cancer Records Linkage Project (CCRLP) ALL Study.
