# Supplementary figures and images for: Heritable variation at the chromosome 21 gene ERG is associated with acute lymphoblastic leukemia risk in children with and without Down syndrome
Source: Leukemia. 2019 Jul 11;33(11):2746–51. doi: 10.1038/s41375-019-0514-9 (PMC6858994; doi:10.1038/s41375-019-0514-9)

**Figure S1**

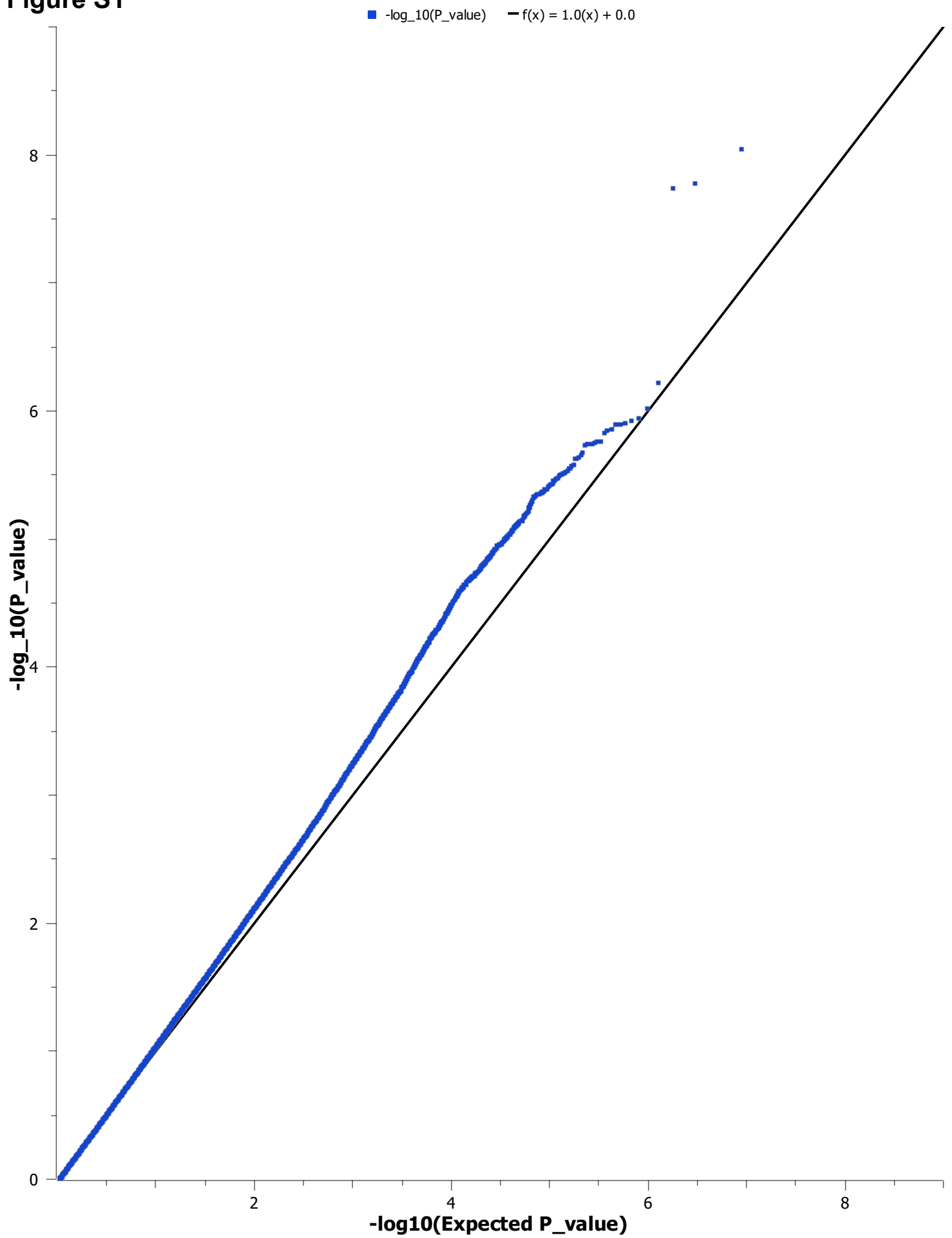

Figure S2

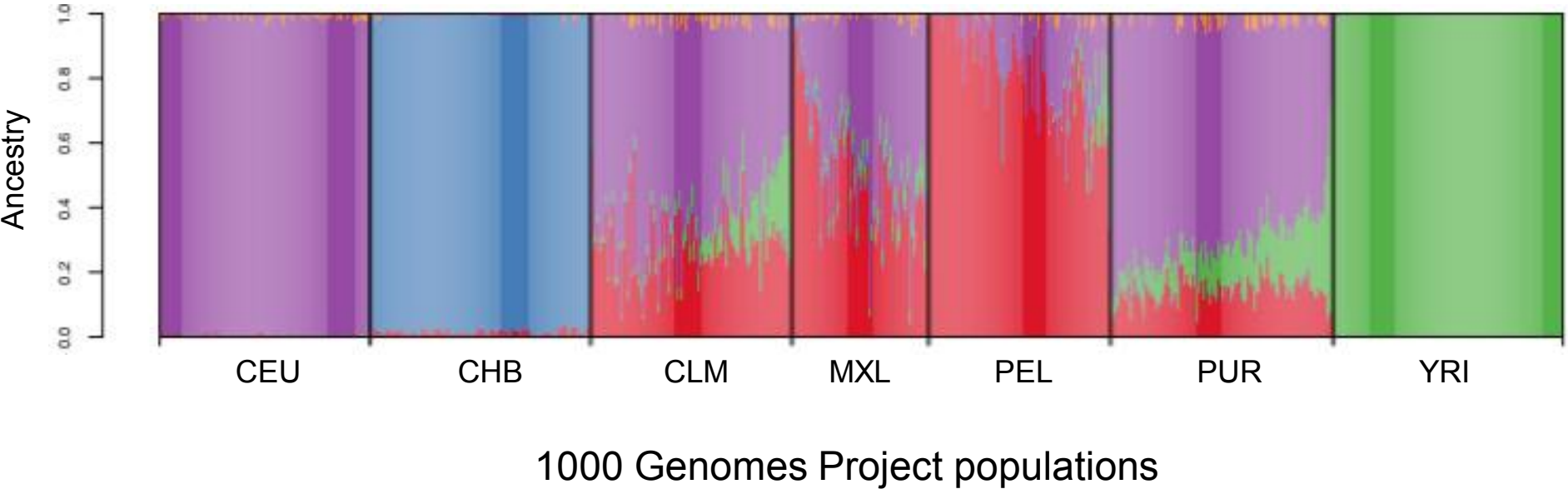

Figure S3

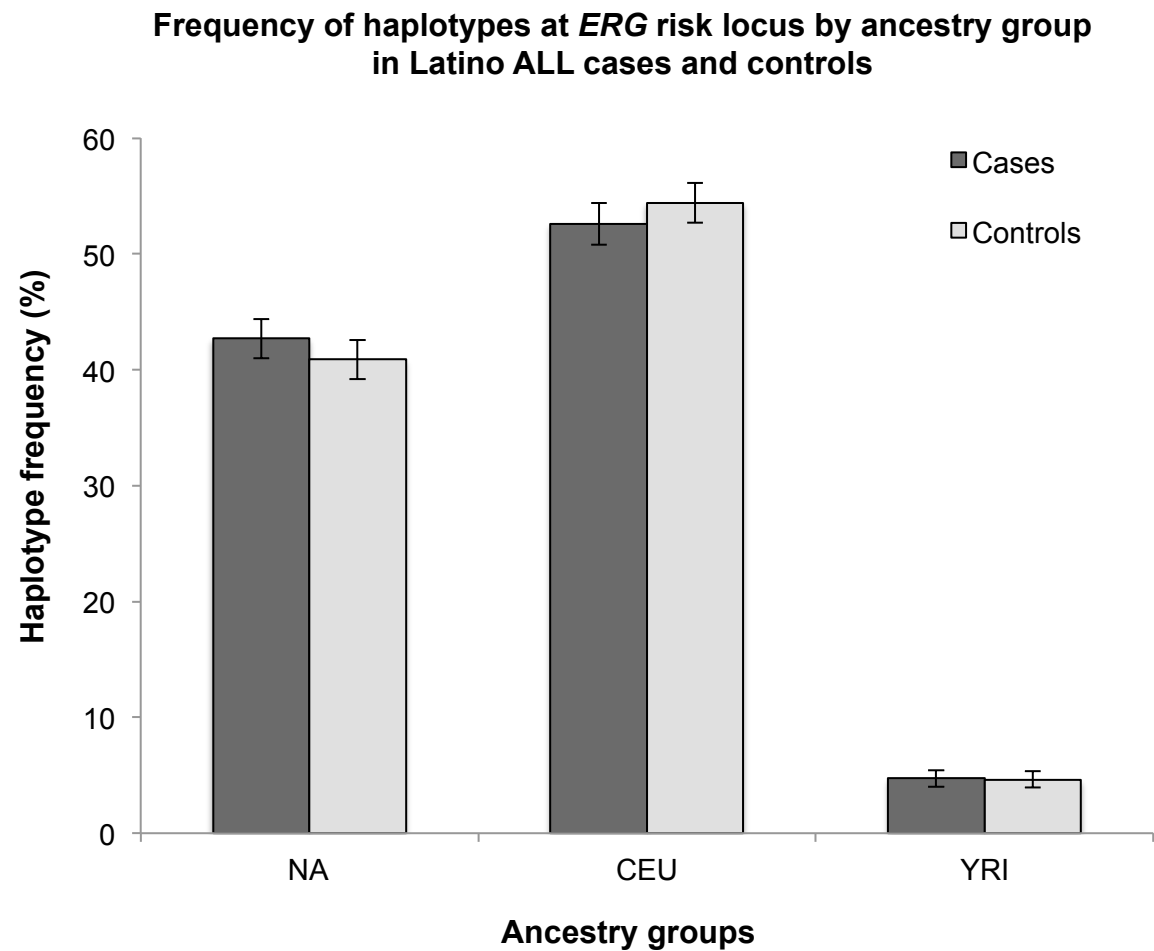

**Figure S4**

**A**

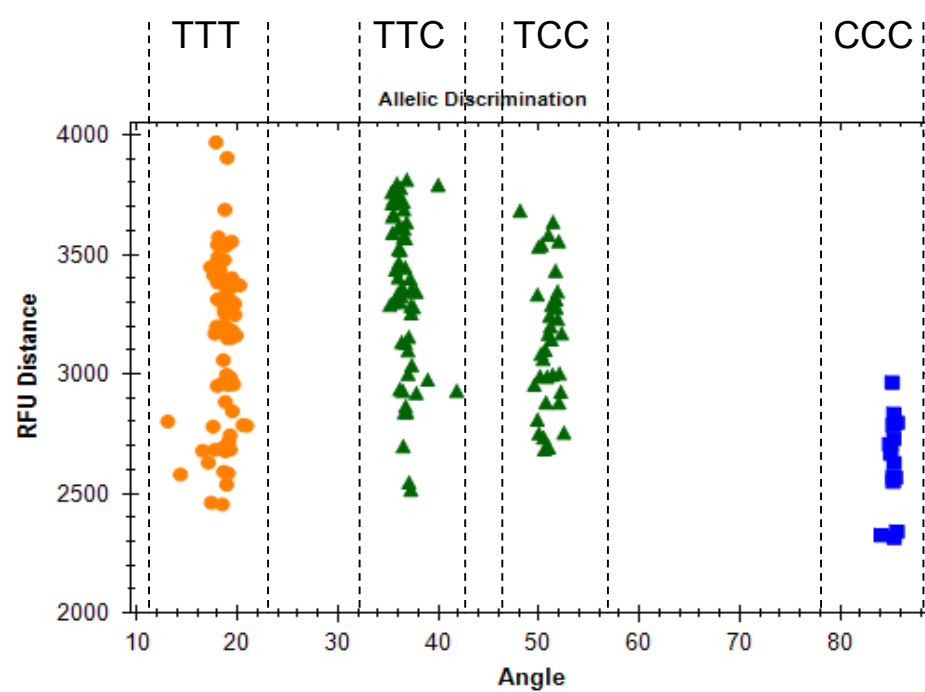

rs2836371 Taqman  
genotype clusters

**B**

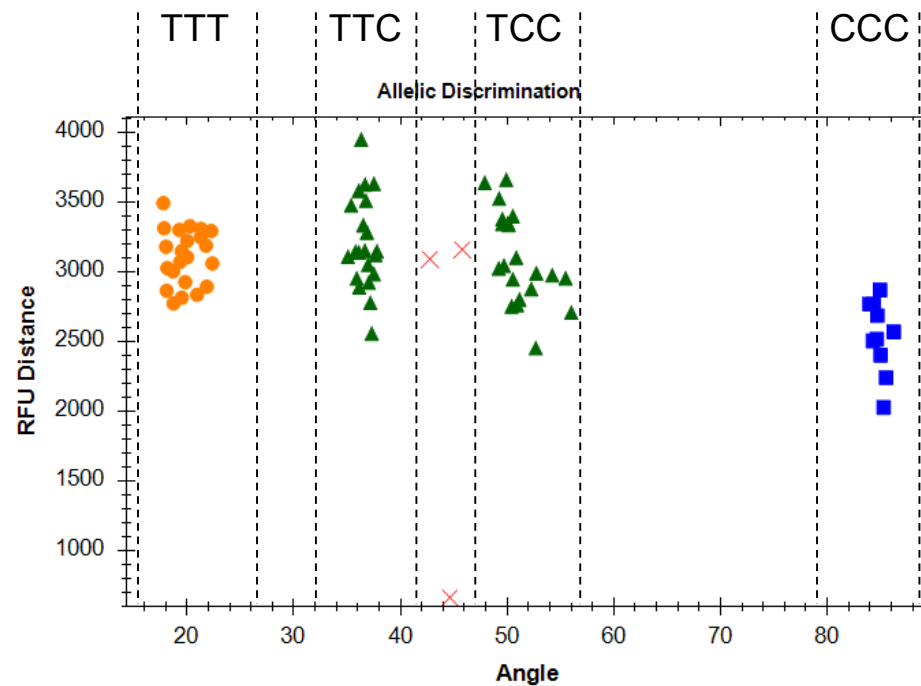

Figure S5

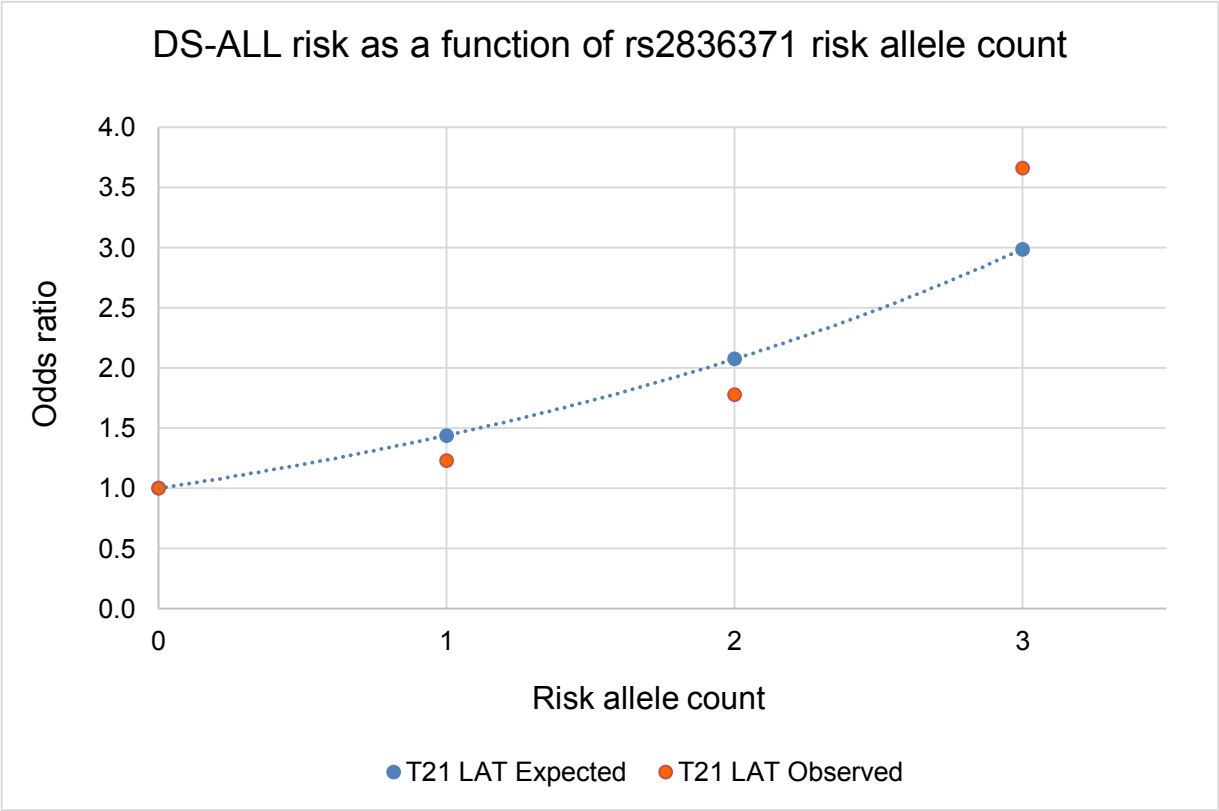

**Figure S6**

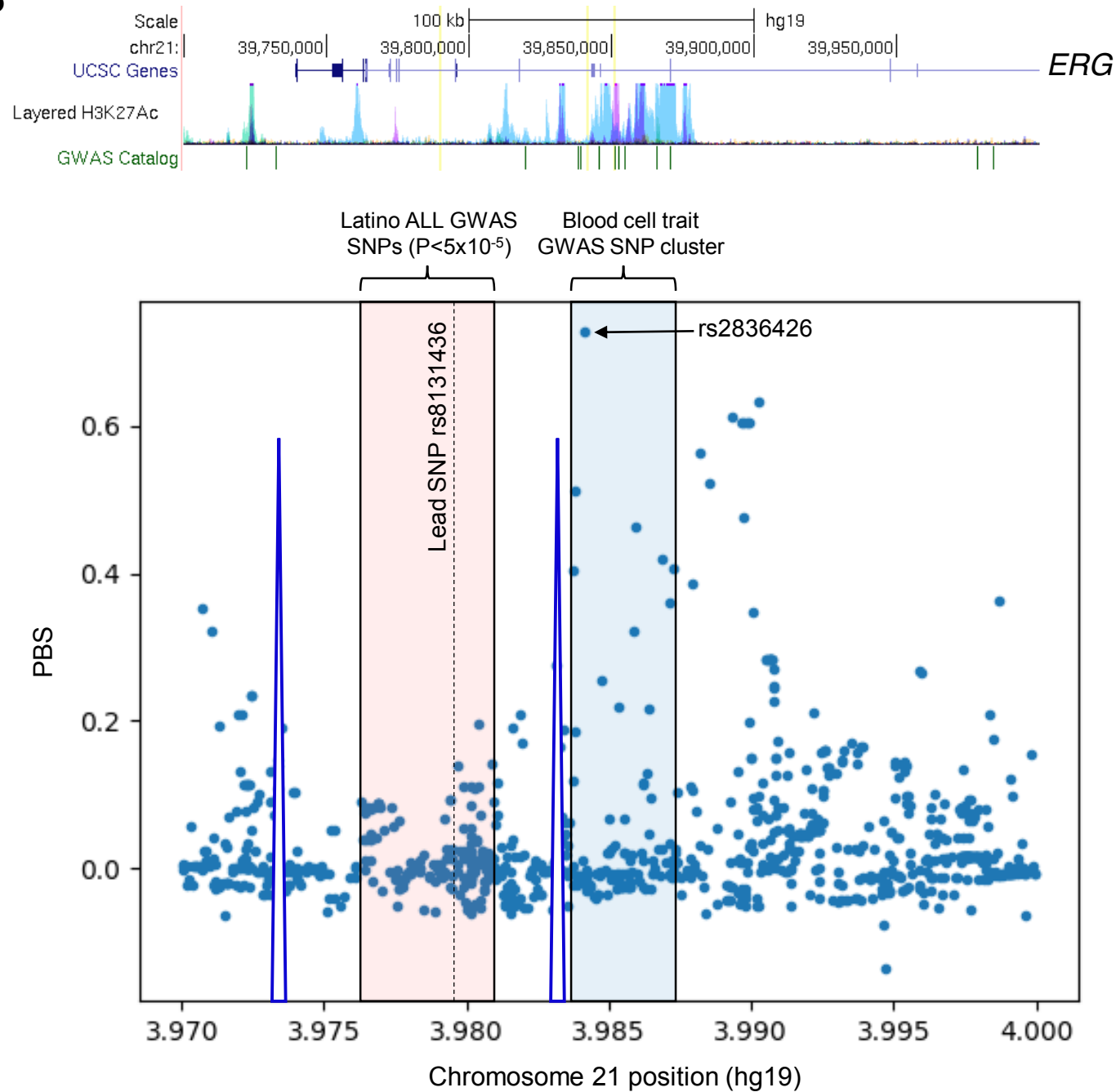

Figure S7

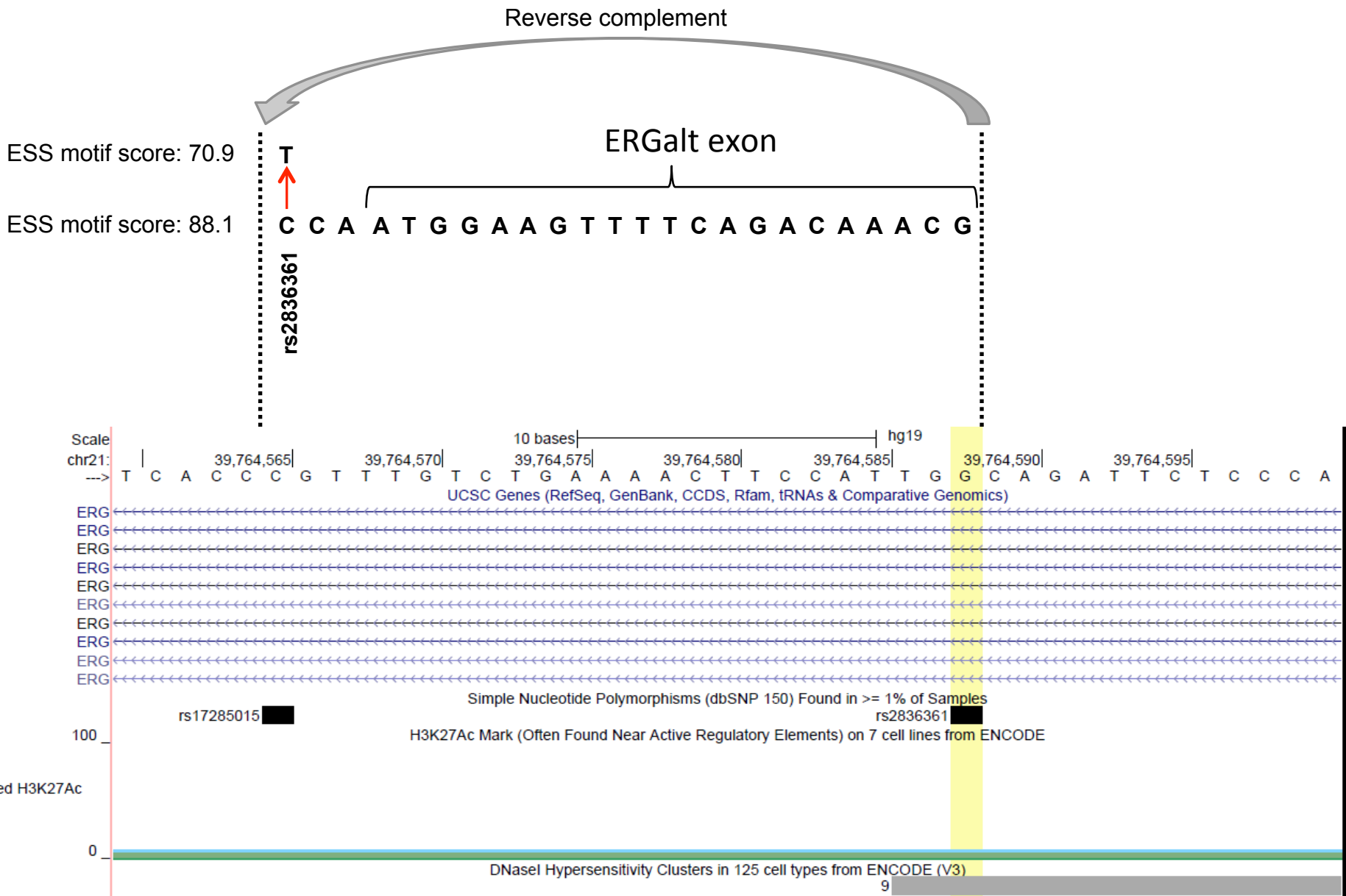

Supplement: Supplementary file 2 — Supplemental Figures [file 41375_2019_514_MOESM2_ESM.pdf]
